# Source Tracker Modeling Based on 16S rDNA Sequencing and Analysis of Microbial Contamination Sources for Pasteurized Milk

**DOI:** 10.3389/fnut.2022.845150

**Published:** 2022-04-28

**Authors:** Bingyao Du, Lu Meng, Haoming Wu, Huaigu Yang, Huimin Liu, Nan Zheng, Yangdong Zhang, Shengguo Zhao, Jiaqi Wang

**Affiliations:** ^1^Key Laboratory of Quality and Safety Control for Milk and Dairy Products of Ministry of Agriculture and Rural Affairs, Institute of Animal Sciences, Chinese Academy of Agricultural Sciences, Beijing, China; ^2^State Key Laboratory of Grassland Agro-Ecosystems, Key Laboratory of Grassland Livestock Industry Innovation, Ministry of Agriculture and Rural Affairs, College of Pastoral Agriculture Science and Technology, Lanzhou University, Lanzhou, China; ^3^Laboratory of Quality and Safety Risk Assessment for Dairy Products of Ministry of Agriculture and Rural Affairs, Institute of Animal Sciences, Chinese Academy of Agricultural Sciences, Beijing, China; ^4^Sericultural and Agri-Food Research Institute, Guangdong Academy of Agricultural Sciences, Key Laboratory of Functional Foods, Ministry of Agriculture and Rural Affairs, Guangdong Key Laboratory of Agricultural Products Processing, Guangzhou, China

**Keywords:** milk, microbial, contamination, 16s rDNA sequencing, Source Tracker

## Abstract

Milk is rich in fat, protein, minerals, vitamins, peptides, immunologically active substances, and other nutrients, and it plays an important role in satisfying human nutrition and health. However, dairy product safety incidents caused by microbial contamination have occurred. We found that the total bacterial numbers in the pasteurized product were low and far below the limit requirements of the food safety standards of the European Union, the United States, and China. At the genus level, the primary microbial groups found in milk samples were *Acinetobacter, Macrococcus, Pseudomonas*, and *Lactococcus*, while in the equipment rinse water and air samples there was contamination by *Stenotrophomonas* and *Acinetobacter*. The Source Tracker model analysis indicated that the microorganisms in the final milk products were significantly related to the contamination in product tanks and raw milk. Therefore, it is the hope that this work can provide guidance to pinpoint contamination problems using the proper quality control sampling at specific stages in the pasteurization process.

## Introduction

Milk, which is a nutrient source for protein, minerals, vitamins, and immunologically active substances, is considered a perfect food and plays an important role in meeting human nutritional needs (1–3). However, it is a good growth media for microorganisms because of the rich nutrients.

Contaminated milk may rapidly deteriorate and pose a potential health threat (4–8). According to reports, the detection rate of pathogens in pasteurized milk in Brazil is 4.7%, and pasteurized milk in China is often reported to be contaminated with pathogens (9–11). These bacteria are not naturally present in the mammary glands of healthy cows, and therefore, it is necessary to track the source of the microorganisms in milk, as they may find their way into milk as a result of poor milking and farming practices (12).

Spanu et al. used polymerase chain reaction (PCR) and pulsed-field gel electrophoresis technology to analyze the contamination sources of *Listeria monocytogenes* in cheese, and found that the processing environment of the cheese factory was the main source of microbial contamination (13). Maya analyzed the source of contamination of methicillin-resistant *Staphylococcus aureus* in pasteurized milk through multilocus sequence typing and pulsed-field gel electrophoresis technology, and found that cross-contamination after processing was one of the main sources of contamination (14). Wang et al. used 16S rRNA-PacBio Single Molecule Real-Time sequencing method to trace the microbes in milk powder and found that raw milk or processing equipment was one of the main sources of contamination (15). These studies are based on the typing and identification of strains based on the culture method or single sequencing technology, which has certain limitations.

In recent years, the Source Tracker modeling based on 16S rDNA sequencing and analysis of microbial contamination sources for raw milk have been widely used. Du et al. used Source Tracker modeling based on 16S rDNA sequencing to analyze the sources of microbial contamination in raw milk and found that the teat liner and teat dip cup were the main sources of contamination (16). Wu et al. used Source Tracker modeling based on 16S rDNA sequencing to analyze the source of microbial contamination in farm milk using automatic milking systems and found that airborne dust was the most important source of contamination (17).

Traceability analysis or determination of the presence of a particular substance or microorganism along a sample processing chain has been widely applied in microbiological studies. However, there have been few studies on the traceability of microorganisms in pasteurized milk and finding the particular processing step in which the contamination most likely occurs. The objective of the current study was to evaluate the traceability of microorganisms in pasteurized milk and analyze the individual processing steps, utilizing the Source Tracker model based on 16S rDNA sequencing, and find the potential sources of microbial contamination, which will help us formulate reasonable disinfection and cleaning procedures to improve the quality of dairy products.

## Materials and Methods

### Sampling

Milk samples were collected at 8 critical control points along the milk processing pathway at the dairy processors in Zhengzhou City, Henan province in May 2019. The ambient temperature was 25°C. The control points included milk tanker (30 tons, raw milk, M1), storage tank (30 tons, raw milk, M2), storage tank for processing (30 tons, raw milk, M3), milk in balance tank (72°C/15 s, M4), milk in balance tank (75°C/15 s, M5), milk in balance tank (80°C/15 s, M6), milk in product tank (72°C/15 s, M7), milk in product tank (75°C/15 s, M8), milk in product tank (80°C/15 s, M9), pasteurized milk (72°C/15 s, M10), pasteurized milk (75°C/15 s, M11), pasteurized milk (80°C/15 s, M12), equipment water rinses (W), and processing factory air (A). The cleaning frequency of the tank was once a day. The milk was stored in the tank for 2 h before processing. A quantity of 200 ml of clean in place (CIP) water from the dairy factory was collected every day, and stored in sterile sampling bottles. For the collection method of air samples, referring to Du et al. (16), milk samples (50 ml) were collected in triplicate each day at each sampling point for 4 consecutive days and immediately stored at −20°C until analysis.

### Detection of Total Viable Bacterial Counts, Alkaline Phosphatase, and Lactoperoxidase

The counts of total viable bacteria(TBC) was determined following the National Standards of the Republic of China (GB 4789.2-2016) (18), alkaline phosphatase was detected following the International Dairy Federation (IDF) procedure (IDF, method 209, 2007) (19), and lactoperoxidase was determined following the Tianjin Dairy Science and Technology Innovation Association Group Standard (T/TDSTIA, method 001, 2021) (20).

### 16S RDNA Sequencing

Milk samples (10 ml each) were centrifuged at 14,000 × g for 5 min, and the precipitate was used for DNA extraction using the HiPure Stool DNA Kits (Magen, Guangzhou, China) according to the manufacturer's instructions. Extracted DNA was amplified using the following PCR cycling program: 94°C for 2 min, followed by 30 cycles at 98°C for 10 s, 62°C for 30 s, and 68°C for 30 s, and a final extension at 68°C for 5 min. The PCR primers were specific for the 16S rDNA V3-V4 region: (5′-3′) 341F: CCTACGGGNGGCWGCAG, 806R: GGACTACHVGGGTATCTAAT (21). PCR reactions utilized KOD Polymerase (Millipore Sigma, Rockville, MD, USA). Reagents were provided with the commercial enzyme kit. DNA recovery of amplicons was obtained following agarose gel electrophoresis using an AxyPrep DNA gel extraction kit (Axygen Biosciences, Union City, AZ, USA). FASTP was used to filter the original data, and reads containing >10% of unknown nucleotides (nt) were deleted as were strings of 20 nt with a mass <50% of the Q value (22, 23). Pairs of clean reads were combined into the original tag with a minimum overlap of 10 nt and a mismatch error of 2%. The original tag sequences were then filtered through QIIME (version 1.9.1) (24) using the reference database v.r20110519 (http://drive5.com/uchime/uchime_download.html) (25) to obtain high quality clean labels. Noise reduction, chimera detection, and aggregation into operational taxonomic units (OTU) containing 97% identities were performed using UPARSE (v. 9.2.64) (26).

The SILVA database (v. 132) (27) was used to classify representative sequences as biological using the naive Bayes model using confidence threshold ranges from 0.8 to 1. Krona (v.2.6) was used to visualize the abundance of each taxonomic group (28). Stacked bar charts were visualized using R project ggplot2 (v.2.2.1) (17, 29) and Omicsmart (http://www.omicsmart.com). Pheatmap software (v. 1.0.12) from Omicsmart was used to indicate species richness.

### Statistical Analyses

The principal coordinate (CAP) graphs were visualized using ggplot2 (30, 31). Pearson correlation coefficient >0.7 was considered significant. Similarly, hierarchical clustering and heat map construction were completed. The Source Tracker modeling based on 16S rDNA sequencing can be used for the analysis of microbial contamination sources for raw milk and pasteurized milk, and can calculate the similarities between different bacterial communities (32). The Source Tracker model was based on the Bayesian algorithm (https://github.com/danknights/sourcetracker, accessed as of July 2021) to predict the composition ratio of the target samples from each source sample, according to the microbial community structure distribution of the target samples and the source samples. The colored sector area indicated the proportion of each source in the samples, and explored the analysis of the source of microbial contamination in the target sample (32–34).

## Results

### Overall Microbiome Analysis

**Figure 1:**
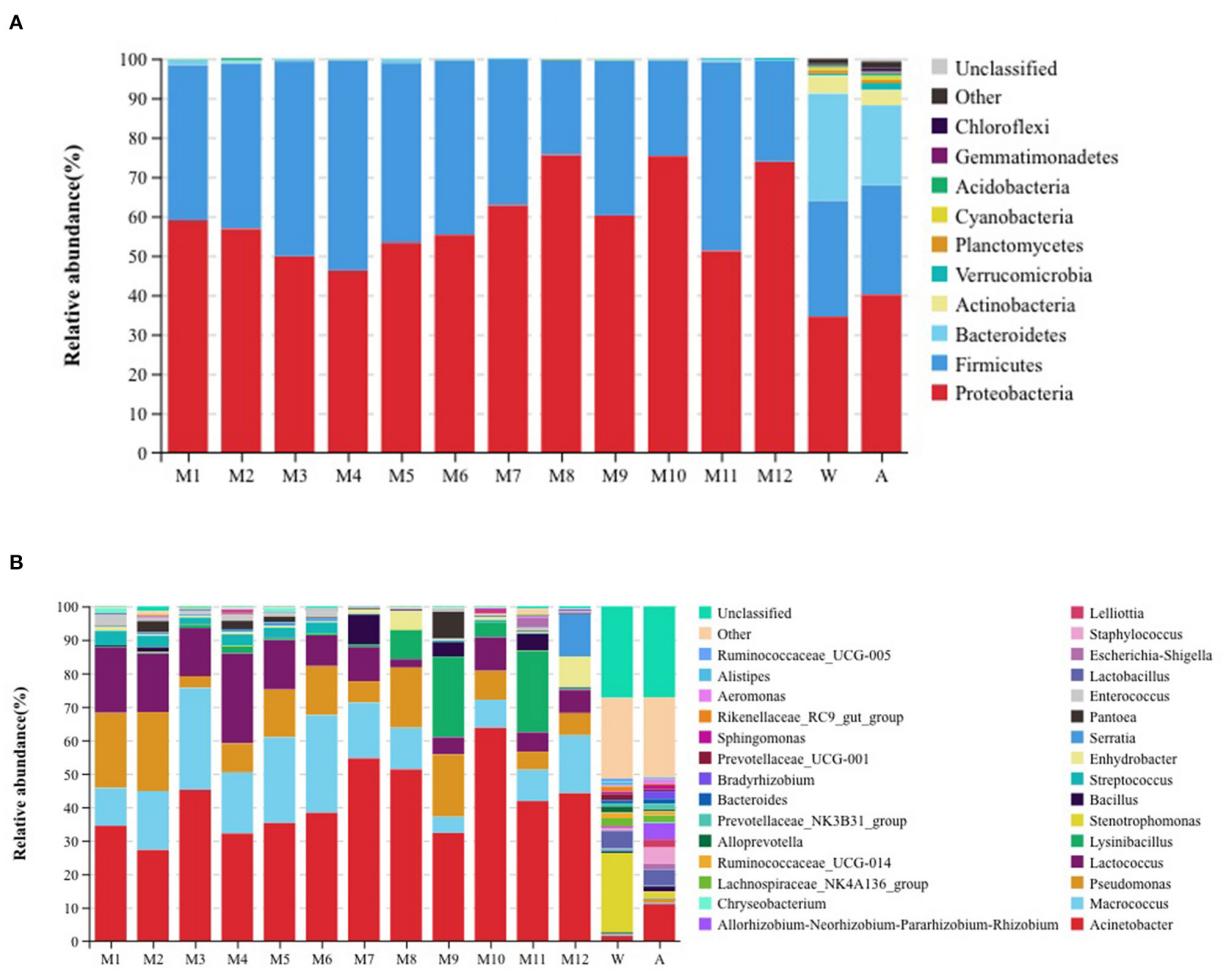
Relative abundance of operational taxonomic units at the **(A)** phylum and **(B)** genus level for the different samples. W: equipment water rinses, A: processing factory air. M1: milk tanker (raw milk); M2: storage tank (raw milk); M3: storage tank for processing (raw milk); M4: milk in balance tank (72°C/15 s); M5: milk in balance tank (75°C/15 s); M6: milk in balance tank (80°C/15 s); M7: milk in product tank (72°C/15 s); M8: milk in product tank (75°C/15 s); M9: milk in product tank (80°C/15 s); M10: pasteurized milk (72°C/15 s); M11: pasteurized milk (75°C/15 s); and M12: pasteurized milk (80°C/15 s).

We tested the total number of colony counts in raw milk and pasteurized product for 4 consecutive days. The average total number of colonies in raw milk was 2.1 × 10^3^ CFU/ml, and the average total number of colonies in the pasteurized products was 2.1 × 10^2^ CFU/ml (72°C for 15 s), 18 CFU/ml (75°C for 15 s), and 2 CFU/ml (80°C for 15 s). The alkaline phosphatase in all pasteurized products was negative (<350 mU/L), and the average lactoperoxidase of the pasteurized products were 6,278.7 U/L (72°C for 15 s), 3,644.2 U/L (75°C for 15 s), and 0 U/L (80°C for 15 s). An analysis of the microbiota of all samples indicated the presence of 10 phyla that were considered as abundant. These included Chloroflexi, Gemmatimonadetes, Actinobacteria, Cyanobacteria,Planctomycetes, Verrucomicrobia, Acidobacteria, Bacteroidetes, Firmicutes, and Proteobacteria. All milk samples had the two major phyla groups of Proteobacteria and Firmicutes. Air and water samples had the largest number of OTUs present, comprising primarily of the three major phyla groups of Proteobacteria, Firmicutes, and Bacteroidetes. A comparison with the raw milk samples indicated that Firmicutes abundance in pasteurized samples gradually decreased while Bacteroidetes almost disappeared, even though Firmicutes and Proteobacteria remained the primary phyla. In addition, the relative abundance of Bacteroidetes in the water and air samples was higher than in the milk samples (Figure 1A). We also found 30 genera that were considered as abundant, of which, *Acinetobacter, Macrococcus, Pseudomonas*, and *Lactococcus* were the most abundant genera found in samples from the milk tanker, storage tanks, storage tank for processing, and milk in balance tank. *Lysinibacillus* was a relatively abundant genus in milk samples from the product tank. *Acinetobacter* was the most abundant genera in pasteurized samples. *Stenotrophomonas* and *Acinetobacter* were the most abundant genera in water and air samples, respectively (Figure 1B). Among the water samples, the relative abundance of *Stenotrophomonas* was higher than in other samples, but the relative abundance of *Acinetobacter* was lower than in other samples.

### Microbiota Diversity Based on Alpha Analysis

**Figure 2:**
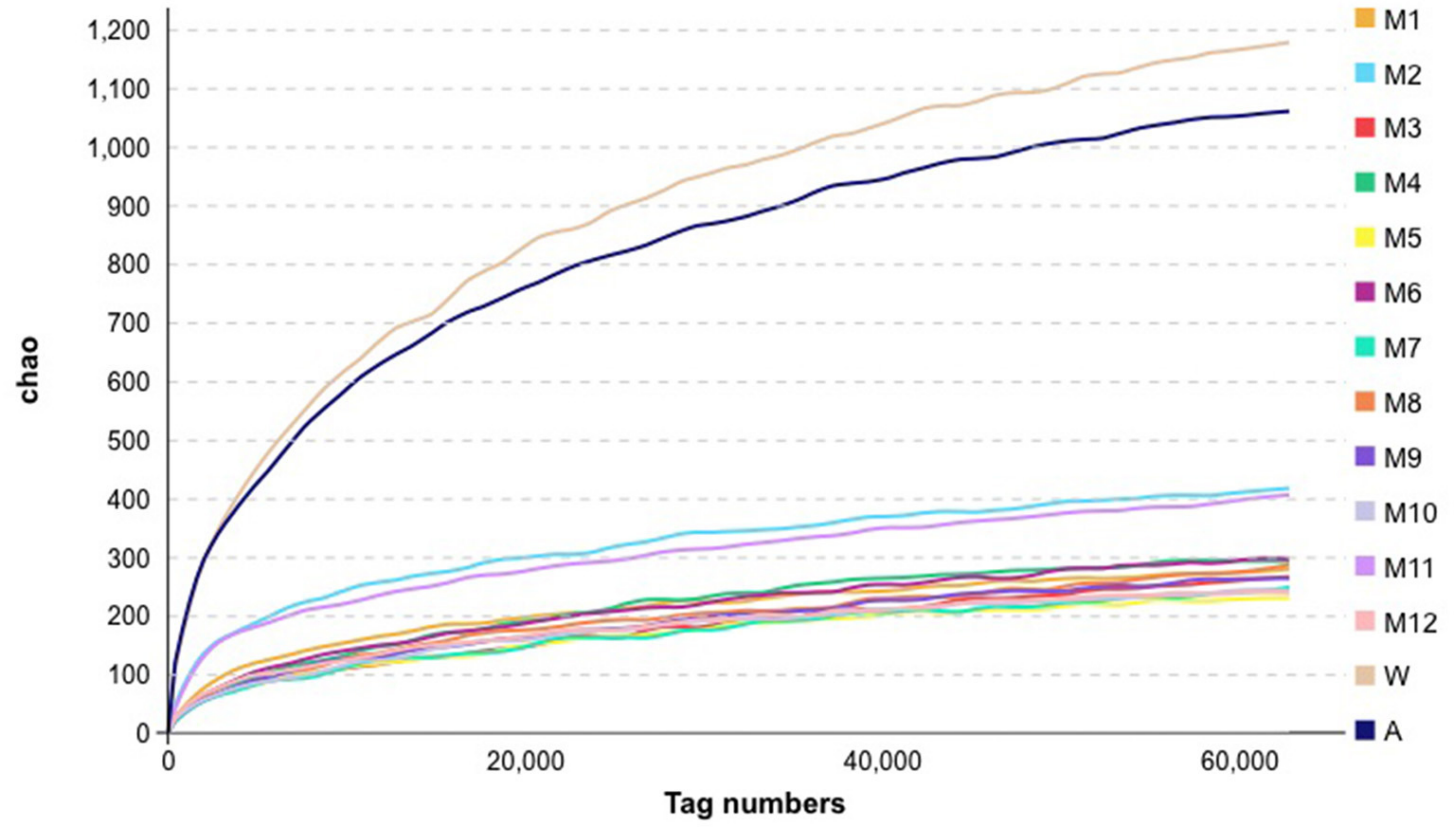
Alpha analysis metrics for bacteria in the samples collected for this study. See Figure 1 for abbreviations.

The diversity within samples and between samples was evaluated using alpha diversity analyses. We found differences in microbiota richness between raw milk in the farm milk tanks and the air samples, when compared with all of the other samples, using the Chao1 index (Figure 2). Compared with raw milk, the Chao1 indices for the samples following pasteurization decreased, indicating a reduction in bacterial abundance.

### Clustering Heat Map Analysis

**Figure 3:**
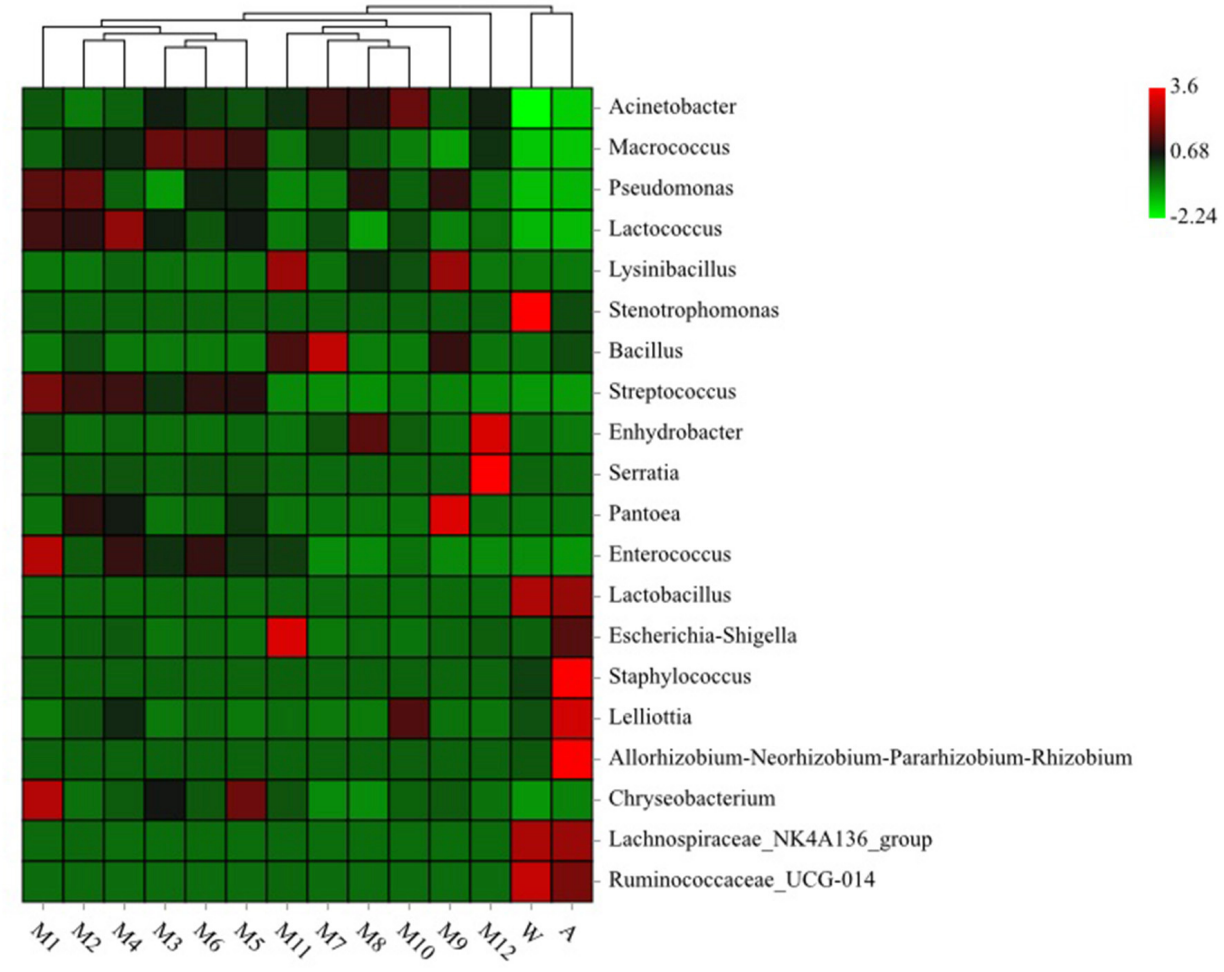
Heatmap analysis for bacteria genera in different samples. See Figure 1 for abbreviations.

We used clustering heat map analysis to further explore the differences in the community structure (the top 20 relative abundances) of microorganisms in different samples at the genus level (Figure 3), and found that milk tanker sample (raw milk), storage tank sample (raw milk), storage tank for processing sample (raw milk), milk in balance tank sample (72°C/15 s), milk in balance tank sample (75°C/15 s), and milk in balance tank sample (80°C/15 s) were apparently clustered together due to *Pseudomonas, Lactococcus, Streptococcus*, and *Enterococcus*. Equipment water rinses sample and processing factory air sample were apparently clustered together due to *Ruminococcaceae*_UCG-014, *Lachnospiraceae*_NK4A136_group, and *Lactobacillus*.

### Canonical Analysis of Principal Coordinates

**Figure 4:**
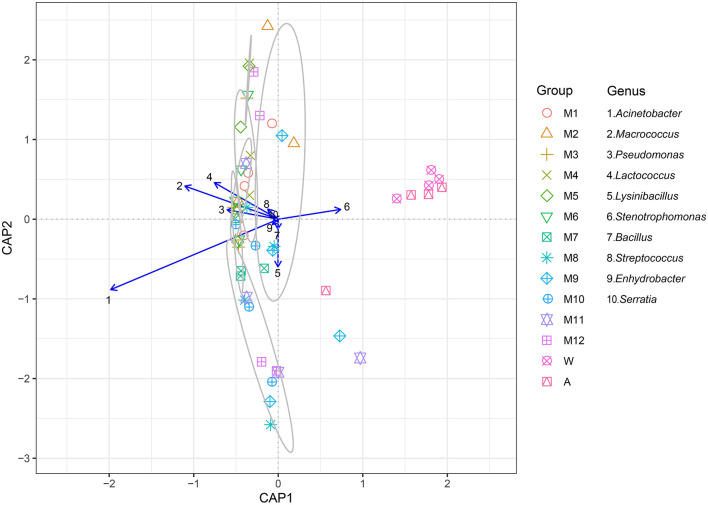
The canonical analysis of principal coordinates (CAP) explained the correlation of the level of the 10 primary bacterial genera (*Acinetobacter, Macrococcus, Pseudomonas, Lactococcus, Lysinibacillus, Stenotrophomonas, Bacillus, Streptococcus, Enhydrobacter, and Serratia*) in different samples. The samples in the gray circle are regarded as a group because the similarity was >70%. See Figure 1 for abbreviations.

Canonical analysis of principal coordinates (CAP) indicated the top 10 most abundant genera (*Acinetobacter, Macrococcus, Pseudomonas, Lactococcus, Lysinibacillus, Stenotrophomonas, Bacillus, Streptococcus*, and *Enhydrobacter*) associated with milk, which were clustered in contrast to the rinse water and the processing factory air represented by *Stenotrophomonas*, which was considered as an independent group with a 70% similarity (Figure 4). *Acinetobacter, Macrococcus, Pseudomonas, Lactococcus*, and *Streptococcus*, which are regarded as typical bacterial taxa of other samples, were not abundant in the processing factory air.

### The Source Tracker Analysis

**Figure 5:**
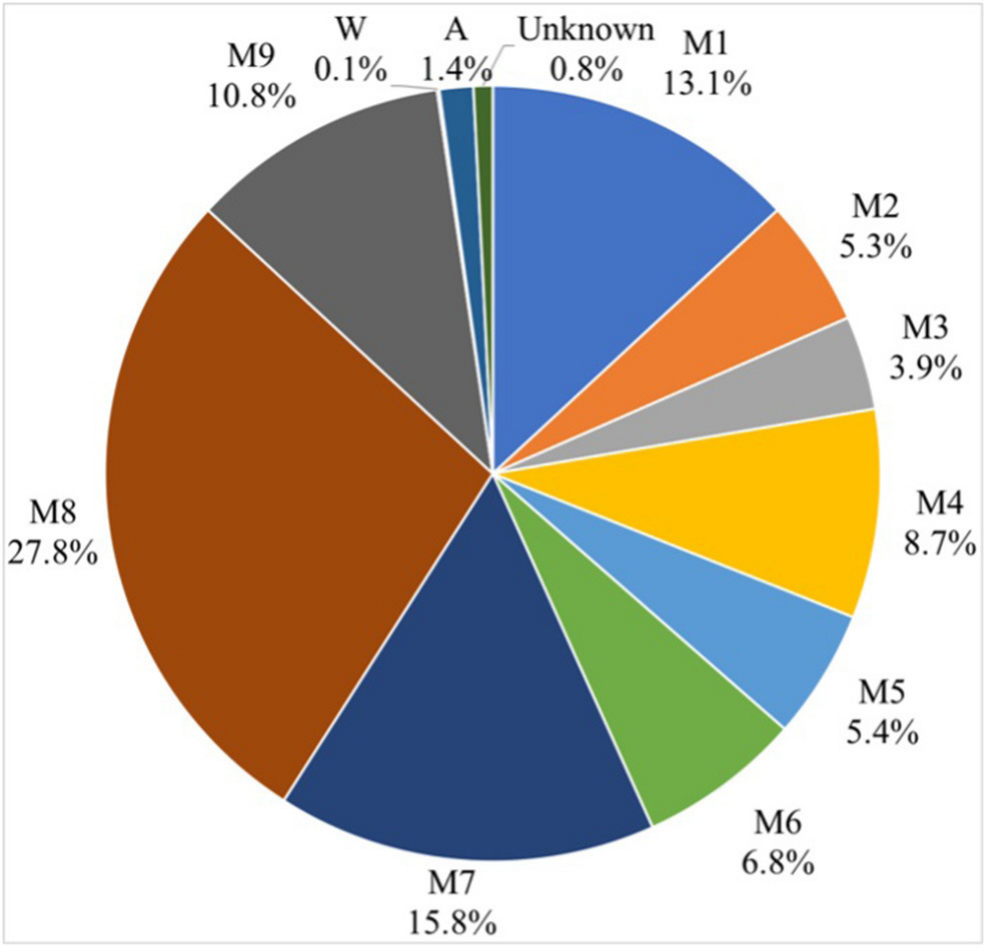
Source Tracker results show the contribution of inferred sources of microbial contamination in dairy products. See Figure 1 for abbreviations.

Sources of environmental contamination in milk were assessed using the Source Tracker model. The sources included all factory environmental samples, raw milk sample, and processed samples, while the sinks were represented by including pasteurized milk samples. The Source Tracker model considered each individual community as a mixed community deposited from other known or unknown environmental sources. The Source Tracker model analysis indicated that the microorganisms in the products were significantly correlated to those in the product tank, followed by milk tanker, which was the second most important source of contaminants (Figure 5).

## Discussion

This study utilized high-throughput sequencing technology to analyze the microbial communities of milk from the delivery tanks to the final stage of pasteurization at a milk processing facility. The primary phyla found in raw milk were *Proteobacteria* and *Firmicutes*, while *Proteobacteria, Firmicutes*, and *Bacteroidetes* predominated the air and equipment rinse water. The *Proteobacteria* was the most dominant phylum in the pasteurized milk samples, followed by *Firmicutes*. After pasteurization of 15 s at 72, 75, and 80°C, the *Proteobacteria* and *Firmicutes* population remained constant. Previous studies have shown that the primary bacterial phyla were *Proteobacteria* and *Firmicutes* (35), while *Pseudomonas, Acinetobacter, Lactococcus, Corynebacterium*, and *Streptococcus* were the most abundant genera in raw milk (36). At the same time, the researchers compared the community structure of microbes in the raw milk collected from two dairy farms and found that *Moraxellaceae, Streptococcaceae, Pseudomonadaceae, Staphylococcaceae*, and *Enterobacteriaceae* were the main microorganisms, but the relative abundance was different. The most obvious were *Enterobacteriaceae* and *Moraxellaceae*. The relative abundances of *Enterobacteriaceae* in the raw milk from the two dairy farms were 82.8 and 0.6%, respectively, and the relative abundances of *Moraxellaceae* in the raw milk of the two dairy farms were 35.7 and 5.4%, respectively (16). The relative abundance of microorganisms in raw milk was different, which may be related to the cleanliness of farm production management, milk tankers, and transport vehicles. *Enterobacteriaceae* and *Pseudomonadaceae* are mainly derived from the environment. Comparing the microbial community structure of the same pasture in summer and winter, the microorganisms with relatively high abundance in summer milk were *Staphylococcaceae, Ruminococcaceae, Aerococcaceae, Lachnospiraceae*, and *Corynebacteriaceae*, while in winter the microorganisms were *Staphylococcaceae, Ruminococcaceae, Aerococcaceae*, and *Lactonospirillaceae*. The microorganisms in raw milk were relatively stable (30). In addition, researchers investigated the microbial community structure from the dairy farm to the final cheese products. Phylum Firmicutes was found as a main phylum, and the ratio between dairy farms and finished cheeses were 31% and 92%, respectively. The relative proportions of *Lactococcus, Lactobacillus, Streptococcus, and Leuconostoc* in cheese products accounts for 69–98% (37). Our results were consistent with another study, in which it was also reported that Gram-negative bacteria were more abundant than Gram-positive bacteria in all samples. Microorganisms in raw milk are mainly *Proteobacteria, Firmicutes, Cyanobacteria, Actinobacteria, Bacteroidetes*, and *Verrucomicrobia*, while *Proteobacteria* and *Firmicutes* are still the dominant phyla in pasteurized milk samples (38). Another study found that the primary bacterial communities in raw milk were *Firmicutes, Actinobacteria, Bacteroidetes, Proteobacteria*, and *Tenericutes* (39). Our study found that similar bacteria dominated the microbial communities of raw milk and pasteurized milk. Therefore, it is necessary to formulate good farm management practices, good production practices, and food safety systems to ensure the safety of dairy products.

Studies of the microorganisms of dairy products have also been reported. For example, Kamimura et al. used 16S rDNA sequencing to analyze the importance of microbial diversity in the processing environment, raw materials and final cheese to the characteristics and quality of cheese. They found that the microbial community structure of raw milk, whey, and environmental samples were significantly different. Both cheese and mature cheese showed a more stable and similar microbial community structure. *Streptococcus* and *Lactococcus* had high relative abundance throughout the cheese processing process (40). Zheng et al. used 16S rDNA sequencing to analyze the diversity of bacterial and fungal communities related to the quality and flavor during cheese maturation, and they found that *Lactobacillus, Streptococcus*, and *Kluyveromyces* were the main microorganisms in cheese (41). Schoen et al. used 16S rDNA sequencing to analyze the microbial community structure of the floor drain and biofilm samples of the Austrian cheese processing plant, and it was found that the microbial community was mainly composed of related bacteria in cheese such as *Lactobacillus* and *Streptococcus thermophilus* (42). Porcellato et al. used 16S rDNA sequencing to analyze the changes in the microbial community structure in raw milk and pasteurized milk, and demonstrated that *Bacillus cereus* was the dominant one in pasteurized milk, compared with pasteurized milk stored at 4°C. In comparison, the diversity of microorganisms was higher in pasteurized milk stored at 8°C (43). In this study, the predominant genera found in the milk sample were *Acinetobacter, Macrococcus, Pseudomonas*, and *Lactococcus*, while *Stenotrophomonas* and *Acinetobacter* were the most abundant genera in air and rinse water. *Macrococcus* are Gram-positive cocci belonging to the *Staphylococcaceae* family and are closely related to *Staphylococcus* but are not considered human pathogens. *Pseudomonas, Lactococcus*, and *Acinetobacter* are known psychrophilic bacteria and are relatively abundant in raw milk (44, 45). In the latter, there are also many species of *Pseudomonas* that produce heat-stable proteolytic and lipolytic enzymes and are responsible for milk quality defects including increased viscosity, sedimentation, aging gelation, fat separation, and increased bitterness. These are the most common reasons for milk spoilage and shortened shelf-life of milk products (46). In addition, *Pseudomonas* are dominant in the microflora of raw milk (38). In our study, *Acinetobacter, Macrococcus, Pseudomonas*, and *Lactococcus* were the most abundant in the microflora of raw milk and products. However, compared with raw milk, the relative abundance of *Pseudomonas* and *Lactococcus* were significantly reduced.

Viazis et al. analyzed the inactivation of specific microorganisms (*Escherichia coli, Listeria monocytogenes, Staphylococcus aureus*, and *Streptococcus agalactiae*) in milk for human consumption through traditional pasteurization, and found that pasteurization of the microorganisms inoculated milk (62.5°C for 30 min) can kill all microorganisms (47). Gabriel et al. analyzed the inactivation of specific microorganisms by pasteurization. The inactivation rate of *Salmonella enterica, Escherichia coli O157:H7, Listeria monocytogenes, Pseudomonas aeruginosa*, and *Staphylococcus aureus* after heating of heavily contaminated milk at 62.5°C for 30 min was 90.8–99.9% (48). Stabel and Lambertz through different pasteurization intensities (i.e., 62.7°C for 30 min, 65.5°C for 16 s, 71.7°C for 15 s, 71.7°C for 20 s, and 74.4°C for 15 s, respectively). Analysis of the inactivation of specific microorganisms (e.g., *Mycobacterium avium* subsp. *paratuberculosis*) found that pasteurization can significantly reduce the survival rate of the organisms, but the inactivation is not complete (49). Heat treatment of milk samples heavily contaminated by pathogenic bacteria shows that traditional pasteurization processes cannot completely eliminate biological hazards. The total number of bacteria in the dairy products in our study was very low and far below the limit requirements of the food safety standards established by the European Union, the United States, and China. We did not detect the presence of any pathogenic bacteria, such as *Salmonella, Staphylococcus aureus*, and *Cronobacter*. This may be related to the improved management methods developed by the dairy farm and processing factory management personnel. For example, the pasture uses sandy bedding, which does not allow bacteria to breed easily. In addition, the cleaning and disinfection procedures during milking also effectively control the raw milk.

The Source Tracker modeling indicated that the microorganisms in the products were significantly correlated to that in the product tank. The product tank becomes a source of microbial contamination due to defects in the management program, e.g., the cleaning-in-place procedures were not followed, the time was too short, rendering these practices ineffective, or the product tank had a secondary contamination source. Our results were consistent with a previous study of bacterial source tracking analysis for milk powder based on 16S rDNA-based single-molecule real-time sequencing technology, indicating that the primary microbes in milk powder originated in the raw milk (15). The Source Tracker model based on 16S rDNA sequencing technology was previously used to analyze potential contamination sources in pasteurized raw milk, and the contributions of cow pasture environmental samples to microbial contamination in raw milk were traced. The teat liners and the teat dip cups were found to be the most important sources of contamination, and this is most likely related to farm cleaning procedures or management practices (16). Source Tracker was also used to analyze the potential sources of microbial contamination in raw milk using an automatic milking system, and the effects of rumen fluid, drinking water, feed, bedding, air, and feces were analyzed. Air was found to be the most important source of contamination. If the cleaning measures of the automatic milking system were complete, the microbial impact on raw milk was relatively small (17).

## Conclusions

In this study, we evaluated the traceability of microorganisms in pasteurized milk, and traced the influence of different steps in the processing lines on the microorganisms in pasteurized milk, using the Source Tracker model based on 16S rDNA sequencing technology. We observed significant differences in richness of microbiota in the samples between the raw milk and the processing factory air samples, even though the uniformity and coverage of bacteria in raw milk and the final products were similar. The Source Tracker model analysis indicated that the microorganisms in the products were significantly related to the product tank. Our analysis can assist in localizing potential sources of microbial contamination and act as guidance for quality control sampling to avoid food quality problems.

## Data Availability Statement

The original contributions presented in the study are publicly available. This data can be found here: Genome Sequence Archive in BIG Data Center, CRA005796.

## Author Contributions

BD: conceptualization, methodology, investigation, and writing—original draft. LM: methodology. HL: data curation. HW and HY: supervision. NZ: conceptualization. YZ and SZ: writing—review, supervision, and funding acquisition. JW: conceptualization and funding acquisition. All authors contributed to the article and approved the submitted version.

## Funding

This study was funded by the Scientific Research Project for Major Achievements of Agricultural Science and Technology Innovation Program (CAAS-ZDXT2019004), the Agricultural Science and Technology Innovation Program (ASTIP-IAS12), and the Modern Agro-Industry Technology Research System of the PR China (CARS-36).

## Conflict of Interest

The authors declare that the research was conducted in the absence of any commercial or financial relationships that could be construed as a potential conflict of interest.

## Publisher's Note

All claims expressed in this article are solely those of the authors and do not necessarily represent those of their affiliated organizations, or those of the publisher, the editors and the reviewers. Any product that may be evaluated in this article, or claim that may be made by its manufacturer, is not guaranteed or endorsed by the publisher.
